# The African Swine Fever Virus Virulence Determinant DP96R Suppresses Type I IFN Production Targeting IRF3

**DOI:** 10.3390/ijms25042099

**Published:** 2024-02-08

**Authors:** Niranjan Dodantenna, Ji-Won Cha, Kiramage Chathuranga, W. A. Gayan Chathuranga, Asela Weerawardhana, Lakmal Ranathunga, Yongkwan Kim, Weonhwa Jheong, Jong-Soo Lee

**Affiliations:** 1 College of Veterinary Medicine, Chungnam National University, Daejeon 34134, Republic of Korea; niranjan3k@gmail.com (N.D.); siniloon@naver.com (J.-W.C.); chathurangakiramage@gmail.com (K.C.); gayachathu123@gmail.com (W.A.G.C.); aselasampath2009@gmail.com (A.W.); lakmalranathunga13@gmail.com (L.R.); 2 Wildlife Disease Response Team, National Institute of Wildlife Disease Control and Prevention, Gwangju 62407, Republic of Korea; kyk5388@korea.kr (Y.K.); purify@korea.kr (W.J.)

**Keywords:** African swine fever virus, DP96R (*UK* gene), IRF3, KPNA

## Abstract

DP96R of African swine fever virus (ASFV), also known as uridine kinase (*UK*), encodes a virulence-associated protein. Previous studies have examined *DP96R* along with other genes in an effort to create live attenuated vaccines. While experiments in pigs have explored the impact of DP96R on the pathogenicity of ASFV, the precise molecular mechanism underlying this phenomenon remains unknown. Here, we describe a novel molecular mechanism by which DP96R suppresses interferon regulator factor-3 (IRF3)-mediated antiviral immune responses. DP96R interacts with a crucial karyopherin (KPNA) binding site within IRF3, disrupting the KPNA-IRF3 interaction and consequently impeding the translocation of IRF3 to the nucleus. Under this mechanistic basis, the ectopic expression of DP96R enhances the replication of DNA and RNA viruses by inhibiting the production of IFNs, whereas DP96R knock-down resulted in higher IFNs and IFN-stimulated gene (ISG) transcription during ASFV infection. Collectively, these findings underscore the pivotal role of DP96R in inhibiting IFN responses and increase our understanding of the relationship between DP96R and the virulence of ASFV.

## 1. Introduction

African swine fever, a highly contagious and fatal viral disease caused by the African swine fever virus (ASFV), is extremely harmful to the swine industry. This viral disease, linked to significant fatality rates in domestic pigs, poses a risk to worldwide pork production and food safety [1,2]. ASFV, the only member of the family *Asfarviridae*, is an icosahedral, enveloped, double-stranded DNA arbovirus (genus *Asfivirus*) with a genome of 170–193 kb [3,4,5]. The virus replicates within the cytoplasm of mononuclear phagocytic cells, and its genome encodes 150–167 proteins that are responsible for viral replication, interaction with host molecules, and regulation of the host innate immune response [6,7]. However, many of these viral proteins have unresolved functions [8,9]; this lack of knowledge impedes our understanding of disease pathophysiology and immune evasion, making vaccine development a challenge. Despite significant research to date, there is no safe and efficient commercial vaccine against ASFV.

Type I IFNs and pro-inflammatory cytokines play a vital role in a host’s defense against invading viruses [10]. The sensor cGAS [cyclic GMP-AMP (2′3′cGAMP) synthase] is primarily responsible for recognizing cytosolic viral DNA [11]. Upon activation, cGAS undergoes a reconfiguration of its catalytic pocket before binding its substrates, adenosine triphosphate (ATP) and guanosine triphosphate (GTP), which are then used to generate the mammalian second messenger 2′3′cGAMP [12,13]. The synthesized 2′3′cGAMP molecule attaches itself to the endoplasmic reticulum (ER) membrane adaptor STING, thereby triggering the structural modifications that are required for its activation [14]. Once activated, STING relocates to the ER–Golgi intermediate compartment, where it recruits and activates TANK-binding kinase 1 (TBK1), which in turn triggers the phosphorylation of interferon regulatory factor 3 (IRF3). Finally, cytoplasmic KPNA molecules recruit phosphorylated and dimerized IRF3 for nuclear translocation; translocated IRF3 then induces the transcription of type I IFNs [15,16]. As a result, to facilitate effective replication within the host, viruses have developed various antagonistic tactics to avoid host type I IFN and inflammatory responses [17,18,19,20]. Previous studies suggest that the virulent ASFV isolate Armenia/07 inhibits generation of IFN-β via the cGAS-STING pathway [21], and that diverse ASFV proteins play various roles in blocking type I IFNs and pro-inflammatory cytokines to facilitate innate immune evasion, which is necessary for the successful replication of ASFV inside macrophages [6,22,23,24,25,26,27,28,29,30,31,32,33,34]. 

ASFV DP96R, the *UK* gene, encodes a 9.6 kDa protein with no significant homology to other known genes in databases. In the late 1990s, a study demonstrated that deleting this highly conserved DP96R from a pathogenic ASFV strain E70 reduced its virulence and protected immunized pigs from challenge with homologous ASFV [35]. Thus, DP96R has been utilized, in combination with other genes, to develop live attenuated vaccines [36,37,38]. While the impact of DP96R on ASFV pathogenicity has been explored in vivo, the underlying molecular mechanism is unknown. Here, we describe a novel mechanism by which DP96R suppresses host type I IFNs and pro-inflammatory reactions by interacting directly with IRF3. Thus, DP96R plays a crucial role in ASFV virulence and could serve as a promising target for the development of live attenuated vaccines.

## 2. Results

### 2.1. DP96R Targets IRF3

**Figure 1:**
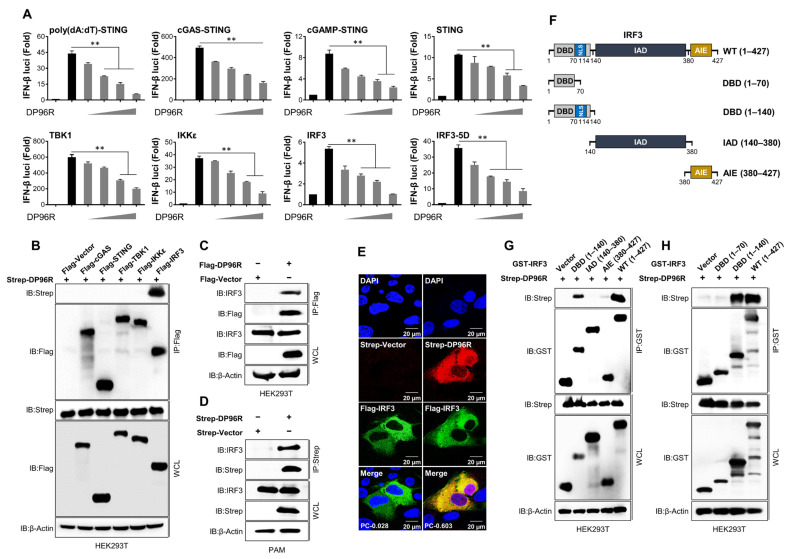
DP96R targets the IRF3 nuclear import domain. (**A**) IFN-β luciferase assay. HEK293T cells were used for STING-, TBK1-, IKKε-, IRF3-, and IRF3-5D-induced IFN-β luciferase assays. HEK293T cells were transfected with Flag-DP96R plasmid, as indicated with relevant stimulants, and firefly luciferase reporter plasmid encoding the IFN-β promoter plus TK-renilla plasmid as transfection control to normalize firefly luciferase activity. Expression plasmids of STING, TBK1, IKKε, IRF3, and IRF3-5D were used as stimulants of the cGAS-STING pathway. STING overexpressing 293-Dual™ hSTING-A162 cells were used for poly(dA:dT)-, cGAS-, and cGAMP-induced luciferase assays. 293-Dual™ hSTING-A162 cells were transfected with Flag-DP96R plasmid as indicated, along with 3×Flag-cGAS expression plasmid, or transfected with poly(dA:dT) or 2′3′cGAMP for 12 h. Thirty six hpt, the luciferase activity of each sample was measured. Results are expressed relative to those renilla luciferases alone. The first and second black bars represent negative and positive controls, respectively. Grey bars represent DP96R dose-dependent transfection. (**B**) HEK293T cells were co-transfected with Strep-DP96R with Flag-tagged cGAS, STING, TBK1, IKKε, IRF3, and Flag control plasmids. Cell lysates were subjected to immunoprecipitation (IP) by Flag antibody, followed by immunoblotting with an anti-Strep and anti-Flag antibodies. (**C**) HEK293T and (**D**) PAMs were transfected with Flag-DP96R and Strep-DP96R and their control plasmids, respectively. Cell lysates were subjected to IP by Flag antibody and Strep beads, followed by immunoblotting with anti-IRF3, anti-Flag or anti-Strep antibodies. (**E**) Colocalization of DP96R and IRF3. PK-15 cells were transfected with Strep-tagged DP96R plasmid and its control plasmid with Flag-tagged IRF3 plasmid, followed by confocal microscopy assay with anti-Flag and anti-Strep primary and anti-mouse (red) and anti-rabbit (green) secondary antibodies. Nuclei were stained with DAPI (blue). (**F**) IRF3 domain constructs. GST-tagged IRF3 wild-type (WT) and amino acid 1–140 (DBD), 140–380 (IAD), 380–427 (AIE), and control vector (GST) were co-transfected to HEK293T cells together with Strep-tagged ASFV DP96R plasmid (**G**). GST-tagged IRF3 WT and amino acid 1–140 (DBD), amino acid 1–70 (DBD excluding NLS), and control vector (GST) were co-transfected to HEK293T cells together with Strep-tagged ASFV DP96R plasmid (**H**). Cell lysates were subjected to GST-PD and immunoblotted with anti-Strep antibodies after immunoblotting the WCL with anti-Strep and anti-GST antibodies. Luciferase data represent three independent experiments, each with similar results, and all the values are expressed as mean ± SD of two biological replicates. All the immunoblot and confocal data represent at least two independent experiments, each with similar results. The scale bar represents 20 μM. Student’s *t*-test: **, *p* < 0.01.

Immune evasion is a critical virulence factor. ASFV uses a wide range of immune evasion strategies to inhibit the generation of IFNs and pro-inflammatory cytokines to enable efficient reproduction within the host. To examine whether ASFV proteins play a role in modulating type I IFN signaling or promoting virus replication, we subjected 158 ASFV viral proteins to individual testing using the Adenoviral replication system. PK-15 cells were transiently transfected with plasmids encoding discrete ASFV genes, or with a control vector (as a negative control). Twenty-four hours post-transfection (hpt), cells were exposed for two hours to a green fluorescent protein (GFP)-expressing adenovirus (ADV-GFP) at a multiplicity of infection (MOI) of 1. Cells infected with the virus were collected 24 h after infection (24 hpi), and the harvested cell pellets were collected to quantify the amount of replicated virus using a fluorescence modulator. As shown in Figure S1, we selected a number of ASFV proteins that increased viral replication. Among them, we identified DP96R. Previous studies have used DP96R in combination with other genes to develop live attenuated vaccines, and the effect of DP96R on the pathogenicity of ASFV has been studied in pigs [35,36,37,38]. However, the exact molecular mechanism underlying virulence remains unknown. Based on our screening results and on previous information, we selected the ASFV *DP96R* gene and used it to determine the precise molecular mechanism that is involved in immune evasion. To explore the crucial role and precise target of ASFV DP96R during type I IFN signaling, we conducted a luciferase promoter assay. This assay involved co-expression of the *DP96R* gene alongside various adapters that are associated with the cGAS-STING pathway. The findings revealed that DP96R inhibited poly(dA:dT)-, cGAS-, 2′3′cGAMP-, STING-, TBK1-, IKKε-, IRF3-, and IRF3-5D (a constitutively active form of IRF3)-mediated activation of the IFN-β promoter in a dose-dependent manner (Figure 1A). This suggests that ASFV DP96R suppresses the cGAS-STING pathway and might target IRF3 to inhibit its dimerization/nuclear translocation or nuclear translocation alone. To confirm the results of the luciferase promoter activity assay, we conducted a series of co-immunoprecipitation (IP) assays to examine the association between ASFV DP96R and IRF3 or several IRF3 deletion mutants (Figure 1B–D,G,H). First, using molecules from the cGAS-STING pathway, we performed an IP assay to determine whether DP96R interacts specifically with IRF3. As shown in Figure 1B, DP96R interacted specifically with IRF3. Furthermore, the DP96R-IRF3 interaction was confirmed by an overexpression IP assay in HEK293T cells (Figure S2A,B). Subsequently, we validated the endogenous interaction between DP96R and IRF3 in HEK293T cells (Figure 1C) and porcine alveolar macrophages (PAMs) (Figure 1D). A Flag-tagged IRF3 and Strep-tagged DP96R overexpression immunofluorescence colocalization assay in PK-15 cells with a significant correlation of 0.603 verified this interaction. It is worth highlighting that ASFV DP96R was localized primarily within the cytoplasm and was not translocated to the nucleus (Figure 1E). 

Next, we constructed expression plasmids containing GST-tagged DBD (a DNA-binding domain spanning aa 1–140), IAD (an IRF association domain spanning aa 140–380), and AIE (an autoinhibition element spanning aa 380–427) domains to identify which of them interact with DP96R (Figure 1F). We found that DP96R precipitated with the DBD of IRF3, but not with the IAD domain (Figure 1G). Thus, we hypothesized that DP96R prevents IRF3 from translocating to the nucleus by inhibiting the IRF3 nuclear localization signal (NLS). The NLS region between IRF3 aa 70–114 comprises two clusters of basic amino acids (K77, R78, and R86, K87) that are embedded in the DBD [39]. Therefore, we removed aa 70–140 from the IRF3-DBD, which includes the NLS region (Figure 1F), and investigated the interaction with DP96R. Exclusion of the NLS impaired the DP96R-IRF3-DBD interaction, confirming that IRF3-NLS is required for the DP96R interaction (Figure 1H). These data strongly suggest that ASFV DP96R hinders type I IFN signaling by interacting with IRF3 and its NLS.

### 2.2. DP96R Impairs the Nuclear Translocation of IRF3

**Figure 2:**
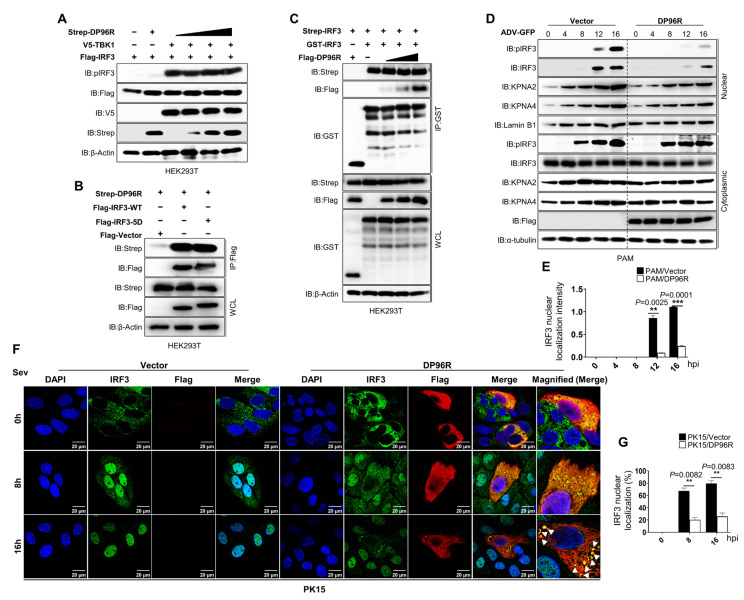
DP96R inhibits the nuclear localization of IRF3. (**A**) IRF3 phosphorylation inhibition assay. HEK293T cells were transfected with Flag-IRF3, and V5-TBK1 with Strep-DP96R dose-dependently. Cell lysates were immunoblotted with anti-pIRF3, -Flag, -V5, and -Strep antibodies. (**B**) HEK293T cells were transfected with Flag-IRF3-WT and Flag-IRF3-5D, and its control vector with Strep-DP96R. Cell lysates were subjected to Flag IP and immunoblotted with anti-Flag and anti-Strep antibodies. (**C**) IRF3 dimerization inhibition assay. HEK293T cells were transfected with Strep and GST-tagged IRF3 with Flag-DP96R dose-dependently. Cell lysates were subjected to GST PD and immunoblotted with anti-Flag, -GST, and -Strep antibodies. (**D**) Cellular fractionation assay. PAMs expressing Flag-DP96R, and its control plasmid were infected with ADV-GFP (1MOI) and harvested at indicated time points. Cytoplasmic and nuclear extracts were then subjected to immunoblot with anti-pIRF3, -IRF3, KPNA2, and KPNA4 antibodies. Lamin B1 and α-tubulin were used to confirm equal loading of proteins of nuclear and cytoplasmic fractions, respectively. (**E**) The histogram represents the relative quantification of the protein levels of the Western blot. Nuclear localization intensity of IRF3 quantified by IRF3 band intensity in nuclear fraction adjusted to nuclear Lamin B1 fraction. (**F**) PK-15 cells were transfected with the Flag control or Flag DP96R plasmid, followed by the Sev (1MOI) infection. Cells were fixed at indicated time points, followed by confocal microscopy assay with anti-IRF3, anti-Flag primary and anti-rabbit (green) and ant-mouse (red) secondary anti-bodies with DAPI (blue) to stain the nuclei. (**G**) By dividing the number of cells expressing nuclear expression of IRF3 by the total number of IRF3-positive cells, the percentages of cells demonstrating nuclear translocation of IRF3 were computed. All the immunoblot and confocal data represent at least two independent experiments, each with similar results. The scale bar represents 20 μM. Student’s *t*-test: **, *p* < 0.01; ***, *p* < 0.001.

Subsequently, to ascertain how ASFV DP96R impacts the activation of IRF3, we examined the phosphorylation, dimerization, and nuclear translocation of IRF3. Different cells express IRF3 constitutively, where it resides within the cytoplasm in an inactive form. Phosphorylation of the C-terminal domain of IRF3 by virus-activated kinases triggers dimerization of IRF3 and its translocation to the nucleus. This event, in turn, leads to the activation of IFN genes [40,41,42]. First, we examined phosphorylation of IRF3 induced by the overexpression of TBK1 in response to escalating quantities of ASFV DP96R protein. Figure 2A shows that increasing doses of ASFV DP96R protein did not affect IRF3 phosphorylation. Furthermore, we examined the interaction between IRF3-5D and DP96R and compared it with the activity of wild-type (WT) IRF3. Concomitant with the results of the IRF3-5D-IFNβ-luciferase assay, and similar to the DP96R-IRF3-WT interaction, DP96R interacted with IRF3-5D (Figure 2B), confirming that DP96R does not interfere with the five amino acid residues of IRF3 that are necessary for its phosphorylation. Second, we performed an IRF3 dimerization assay using IRF3 plasmids tagged with Strep and GST and exposed them to increasing doses of Flag-tagged DP96R. Again, DP96R did not affect IRF3 dimerization (Figure 2C). Third, we performed an ADV-GFP-induced IRF3 nuclear translocation assay using DP96R and a control vector stably expressing PAMs. Cytoplasmic and nuclear fractions were obtained from harvested PAMs, and immunoblotting was carried out to identify specific proteins. The nuclear uptake of IRF3 by DP96R-expressing PAMs was diminished significantly (Figure 2D,E). At 16 hpi, nuclear translocation of IRF3 in DP96R-expressing PAMs was impaired by nearly ~80% compared with that in control PAMs. Even though DP96R does not target IRF3 phosphorylation, DP96R-expressing PAMs showed suppression of phosphorylated IRF3 in the cytoplasmic fraction only at 16 h, which might be due to the inhibition of the IFN signaling pathway by nearby bystander cells. Suppression of type I IFN reduces the transcription of antiviral genes that affect the activation of the cGAS-STING axis. To further confirm the suppressive impact of ASFV DP96R on type I IFN signaling, we carried out an immunofluorescence assay in PK-15 cells overexpressing DP96R or control plasmids after infection with Sendai virus (Sev) at specified time intervals. Upon viral infection, the quantity of nuclear-translocated IRF3 in control cells at 16 hpi increased to nearly ~80% of the total cellular level. By contrast, the amount of IRF3 within the nucleus of PK-15 cells expressing ASFV DP96R was nearly ~30% of the total cellular level (Figure 2F,G). At each time point, DP96R colocalized with endogenous IRF3, and the interaction was strongest at 16 hpi. These findings indicate that ASFV DP96R disrupts the nuclear translocation of IRF3, leading to a reduction in type I IFN production.

### 2.3. DP96R Disrupts Type I IFN Signaling and Subsequent Transcription of Antiviral Genes

**Figure 3:**
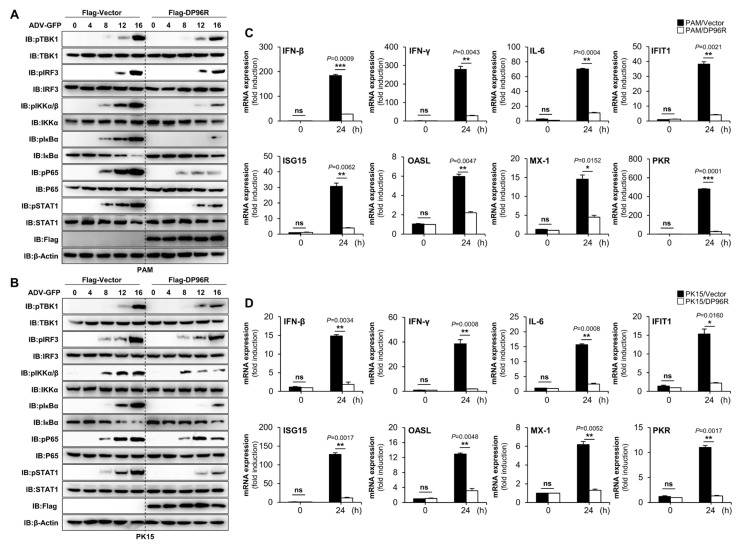
DP96R impairs cGAS-STING signaling and transcription of antiviral genes. Flag-tagged DP96R stably expressing PAMs (**A**) and transfected PK-15 cells (**B**) were infected with ADV-GFP (1MOI), and cells were harvested at indicated time points. DP96R protein expression level and total and phosphorylated TBK1, IRF3, IKKα (pIKKα/β), IκBα, P65, and STAT1 were measured by immunoblotting. β-actin was used as a loading control indicator. PAMs (**C**) and PK-15 cells (**D**), expressing Flag-DP96R with Flag control, were mock-infected and infected with Adenovirus GFP (1MOI), and following 0 hpi and 24 hpi, total RNA was extracted at indicated time points. Quantitative RT-PCR analyzed the mRNA transcripts of the indicated genes relative to internal control of porcine GAPDH. All qRT-PCR data represent at least two independent experiments, each with similar results, and the values are expressed as the mean ± SD of two biological replicates. All the immunoblot data represent at least two independent experiments, each with similar results. Student’s *t*-test: *, *p* < 0.05; **, *p* < 0.01; ***, *p* < 0.001; ns, not significant.

To conduct a more thorough assessment of the effects of DP96R on the virus-induced type I IFN signaling pathway, we examined the phosphorylation status of TBK1, IRF3, IKKα/β, IκBα, P65, and STAT1 in PAMs stably expressing DP96R, as well as in PK-15 cells that were transfected with DP96R. ADV-GFP-infected cells were harvested at the specified time points. Specific antibodies were used to assess the phosphorylation status of signaling molecules involved in the cGAS-STING signaling axis, the NF-κB signaling axis, and the type I IFN pathway that is associated with virus-related processes. The levels of TBK1, IRF3, IKKα/β, IκBα, P65, and STAT1 phosphorylation were notably reduced in PAMs expressing DP96R (Figure 3A) and in PK-15 cells (Figure 3B) compared with control cells. Subsequently, to clarify the impact on IFN signaling and the IFN-antagonistic function of ASFV DP96R, we examined the transcription of genes related to IFN (*IFN-β* and *IFN-γ*), a pro-inflammatory cytokine (*IL-6*), and IFN-stimulated genes (ISGs) *IFIT1*, *ISG15*, *OASL*, *MX-1*, and *PKR*. For this experiment, PAMs stably overexpressing DP96R and PK-15 cells transiently overexpressing DP96R, both of which had been transiently transfected, were subsequently infected with ADV-GFP, followed by quantitative real-time PCR (qRT-PCR) using specific primers for the genes of interest (Table S1). Compared with control cells, DP96R-overexpressing PAMs (Figure 3C) and PK-15 cells (Figure 3D) showed a marked reduction in their expression of mRNA encoding *IFN-β*, *IFN-γ*, *IL-6*, and other antiviral genes.

These findings indicate that the interaction of DP96R with IRF3 has a detrimental impact on host type I IFN signaling pathways, thereby suppressing the transcription of antiviral genes.

### 2.4. DP96R Negatively Regulates Innate Immune Responses against Viral Infection

**Figure 4:**
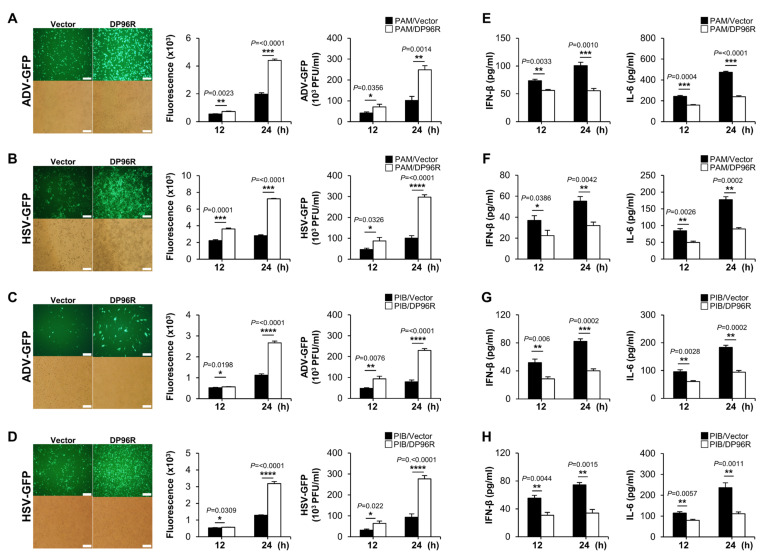
DP96R impairs virus-induced innate immune responses. Stably expressing Flag-DP96R protein in PAMs and PIBs with Flag-control cells were infected with ADV-GFP (1MOI) (**A**,**C**) and HSV-GFP (1MOI) (**B**,**D**). Viral replication was determined at 24 hpi by GFP expression levels by fluorescence microscopy and quantified at 12 hpi and 24 hpi by a fluorescence modulator. The virus titers of each sample were determined by plaque assay in A549 cells and Vero cells. Porcine IFN-β and IL-6 secretion in cell culture supernatant at 12 hpi and 24 hpi were determined by ELISA (**E**–**H**). Data represent at least two independent experiments, each with similar results, and the values are expressed as mean ± SD of three biological replicates. The scale bar represents 50 μM. Student’s t-test: *, *p* < 0.05; **, *p* < 0.01; ***, *p* < 0.001; ****, *p* < 0.0001.

So far, the data imply that ASFV DP96R inhibits type I IFN signaling and transcription of antiviral genes by targeting the nuclear translocation of IRF3. To investigate the role of DP96R during DNA virus replication in vitro, we infected PAMs stably expressing Flag-tagged DP96R or control vector, porcine immortalized bone marrow-derived macrophages (PIBs), and monkey kidney epithelial cells (MA-104) with three GFP proteins expressing DNA viruses. Additionally, we transfected PK-15 cells with a Flag-tagged DP96R or control vector, followed by infection with a DNA virus. Cells were infected with three IFN-sensitive surrogate DNA viruses expressing GFP protein (ADV-GFP, herpes simplex virus (HSV-GFP), and vaccinia virus (VACV-GFP)) instead of ASFV [43,44,45]. The efficacy of transient transfection with the plasmids and the expression of DP96R protein in each cell line was validated by immunoblotting (Figure S3A–D). Remarkably, virus replication was more robust in PAMs, porcine epithelial cells, and the monkey cell line overexpressing DP96R than in control cells (Figure 4A–D, Figures S4A–D and S5A–D). Next, we conducted ELISAs to determine the amounts of IFN-β and IL-6 that were secreted by each virus-infected cell line. In alignment with the outcome of the viral replication test, we observed that DP96R-overexpressing cells secreted lower levels of cytokines than control cells (Figure 4E–H, Figures S4E–H and S5E–H). In line with the earlier findings, both the virus replication and ELISA assays confirmed that DP96R inhibited production of type I IFNs and pro-inflammatory cytokines. As a result, the replication of viral DNA in porcine macrophages, epithelial cells, and monkey epithelial cells is increased.

IRF3 plays a crucial role in multiple innate immune signaling pathways, including RIG-I-like Receptor (RLR) signaling, cGAS/STING signaling, and TLR3 signaling [46]. Viral-derived RNAs trigger RLR signaling, resulting in the activation of IRF3 [47,48]. Thus, we used two RNA viruses, the GFP-expressing Newcastle disease virus (NDV-GFP) and the PR8 strain of H_1_N_1_ virus (PR8-GFP), to infect PAMs and MA104 cells stably expressing DP96R protein. As expected, both cell lines expressing ASFV DP96R protein showed a significant increase in GFP expression and virus replication relative to control cells (Figure S6A–D). Subsequently, we quantified the amounts of IFN-β and IL-6 that was secreted by each virus-infected cell line. Consistent with the virus replication results, the ELISAs showed that cells stably expressing DP96R secreted lower amounts of IFNs and cytokines than control cells (Figure S6E–H). These findings reinforce the idea that DP96R promotes viral replication by interacting with IRF3, thereby exerting a negative regulatory effect on the production of type I IFN.

### 2.5. DP96R Inhibits Interaction between IRF3 and KPNA

**Figure 5:**
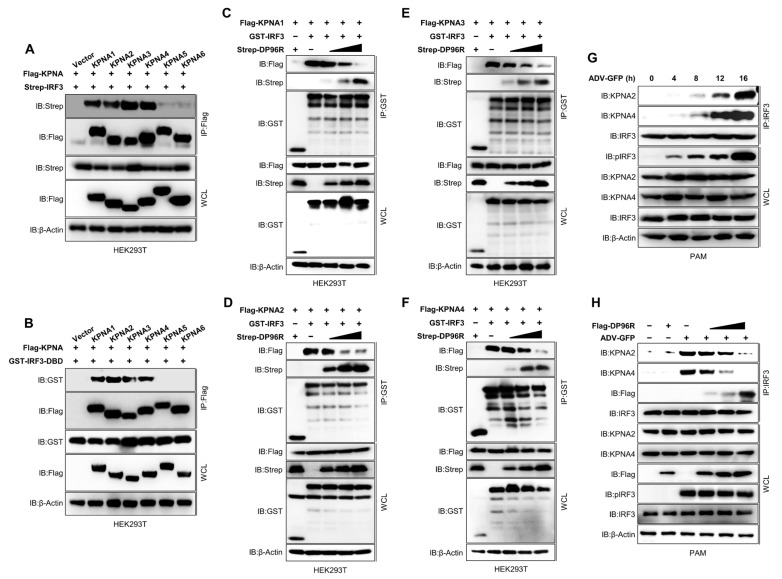
DP96R impairs IRF3-KPNA interaction. (**A**) HEK293T cells were transfected with Flag-KPNA1-KPNA6 and its control plasmid along with the Strep IRF3 plasmid. Cell lysates were subjected to Flag IP and immunoblotted with anti-Strep and anti-Flag antibodies. (**B**) HEK293T cells were transfected with Flag-KPNA1-KPNA6, its control plasmid, and GST-IRF3-DBD mutant plasmid. Cell lysates were subjected to Flag IP and immunoblotted with anti-Flag and anti-GST antibodies. (**C**–**F**) KPNA-DP96R competition assay. HEK293T cells were transfected with (**C**) Flag-KPNA1, (**D**) KPNA2, (**E**) KPNA3, (**F**) KPNA4, GST IRF3, its control, and Strep DP96R plasmids in a dose-dependent manner. Cell lysates were subjected to GST PD and immunoblotted with anti-Flag, -Strep, and -GST antibodies. (**G**) PAMs were infected with ADV-GFP (1MOI), and cells were harvested at indicated time points. Cell lysates were subjected to IRF3 IP and immunoblotted with anti-KPNA2, -KPNA4, -pIRF3, and -IRF3 antibodies. (**H**) PAMs were transfected with Flag-DP96R dose-dependently and infected with ADV-GFP (1MOI), and cells were harvested at indicated time points. Cell lysates were subjected to IP with IRF3 antibodies and immunoblotted with anti-KPNA2, -KPNA4, -pIRF3, -IRF3, and -Flag antibodies. Data represents at least two independent experiments, each with similar results.

To explore the mechanism by which DP96R hinders the nuclear translocation of IRF3, we examined the interaction between karyopherin α (KPNA) and IRF3 in the presence of DP96R. Proteins that have an NLS are shuttled from the cellular cytoplasm to the cellular nucleoplasm by KPNA, which recognizes the NLS of the cargo protein and recruits the KPNB subunit for nuclear localization [49,50]. Many virus proteins block the cargo-NLS-KPNA interaction to inhibit the movement of transcription factors into the cell nucleus, thereby impairing secretion of type I IFNs and pro-inflammatory cytokines [29,51,52]. Previous studies demonstrated that KPNA3 and KPNA4 interact with IRF3 [53,54,55], whereas a recent study revealed that of the six KPNAs, only KPNA2 interacts with IRF3 [56]. Therefore, we next examined the interaction between IRF3 and KPNA1-KPNA6. Interestingly, we found that KPNA1, KPNA2, KPNA3, and KPNA4 interacted strongly with IRF3 (Figure 5A). Next, we checked the interaction between KPNA and the DBD of IRF3, including the NLS region. As shown in Figure 5B, KPNA1, KPNA2, KPNA3, and KPNA4 interacted strongly with the IRF3-DBD. Accordingly, we hypothesized that, despite the slight differences in sequence homology, KPNA1, KPNA2, KPNA3, and KPNA4 interact with a similar motif within the IRF3-NLS region. Next, we performed a competition binding assay in which we overexpressed KPNA1–KPNA4, IRF3, and DP96R in HEK293T cells in a dose-dependent manner. Then, we examined the IRF3-DP96R interaction and the KPNA-IRF3 interaction. An increase in the interaction between DP96R and IRF3 coincided with a reduction in the interaction between KPNA and IRF3 (Figure 5C–F). These results suggest that the DP96R and IRF3 interaction interferes with the KPNA-IRF3 interaction in a dose-dependent manner. The inhibition of the IRF3-KPNA interaction in this overexpression system was verified under virus-induced conditions by performing a competition assay in PAMs stably expressing DP96R. In the first step, we determined the optimal time point of the association between cellular IRF3 and KPNA2 and KPNA4 in PAMs. PAMs were exposed to ADV-GFP (MOI = 1) and then collected at specified intervals. Cellular IRF3 was pulled down and immunoblotted with anti-KPNA2 and anti-KPNA4 antibodies. Figure 5G shows that the ADV-GFP infection-induced interaction between KPNA2 and KPNA4 with IRF3 was strong at 16 hpi, at which point the phosphorylation of IRF3 was augmented (later time points were not tested). Next, we used an endogenous competition binding assay using PAMs that were transiently transfected with DP96R or empty vector, followed by infection with ADV-GFP. As the interaction between DP96R and IRF3 increased, the interaction between IRF3 and KPNA2 or KPNA4 decreased (Figure 5H). 

### 2.6. DP96R Interacts with the Major KPNA-Binding Site within IRF3

**Figure 6:**
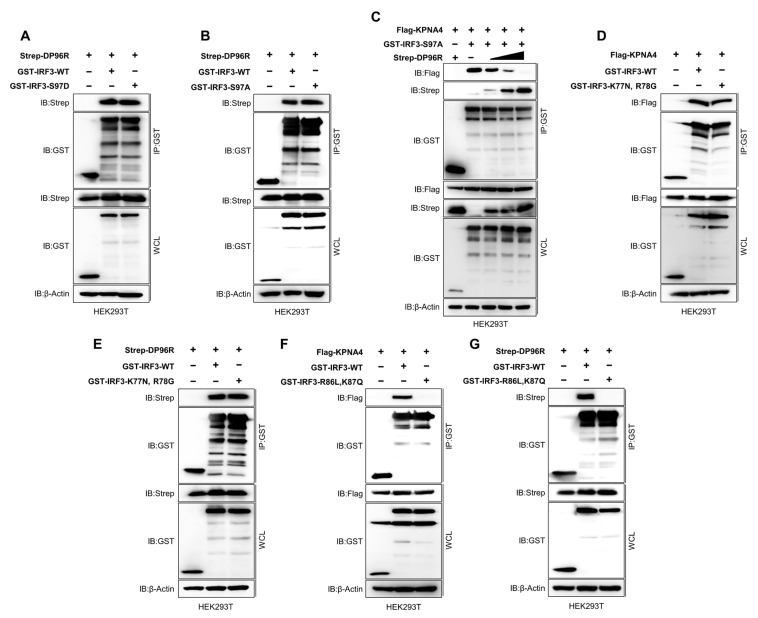
DP96R interacts with the KPNA major binding site of IRF3. HEK293T cells were transfected with GST-IRF3-WT, GST-IRF3 (S97D) (**A**) or GST-IRF3 (97A) (**B**), and Strep-DP96R plasmid. Cell lysates were subjected to GST PD and immunoblotted with anti-Strep, and -GST antibodies. (**C**) KPNA4-DP96R competition assay. HEK293T cells were transfected with Flag-KPNA4, GST-IRF3 (S97A), its control, and Strep DP96R plasmid in a dose-dependent manner. Cell lysates were subjected to GST PD and immunoblotted with anti-Flag, -Strep, and -GST antibodies. (**D**,**E**) HEK293T cells were transfected with GST-IRF3-WT, GST-IRF3 (K77N, R78G), its control plasmid, and Flag-KPNA4 (**D**) or Strep-DP96R (**E**) plasmid. Cell lysates were subjected to GST PD and immunoblotted with anti-Flag, -Strep and -GST antibodies. (**F**,**G**) HEK293T cells were transfected with GST-IRF3-WT, GST-IRF3 (R86L, K87Q) and its control plasmid along with Flag-KPNA4 (**F**) or Strep-DP96R (**G**) plasmid. Cell lysates were subjected to GST PD and immunoblotted with anti-Flag, -Strep, and -GST antibodies. Data represents at least two independent experiments, each with similar results.

Previously, Shun Li et al. reported that dephosphorylation of serine 97 (S97) within IRF3 is critical for the nuclear translocation of IRF3 [57]. Upon virus infection, nuclear translocation of IRF3 was inhibited by the S97D substitution in IRF3 (which imitates the phosphorylated condition) in IRF3- and IRF7-knockout cells. Zeng Cai et al. also showed that the S97D substitution disrupts the KPNA2-IRF3 interaction, whereas IRF3 S97A (which mimics the dephosphorylated state) triggers the interaction. These findings imply that when IRF3 is dephosphorylated at S97, it strengthens the interaction with KPNA2, thereby facilitating the nuclear translocation of IRF3 [56]. To determine how DP96R interferes with the IRF3-KPNA interaction, we performed a series of binding assays using several mutant IRF3 plasmids. First, we asked whether DP96R prevents the dephosphorylation of IRF3 at position S97 to inhibit nuclear translocation. To this end, we confirmed that the S97D and S97A substitutions in IRF3 inhibit and enhance the interaction with KPNA4, respectively (Figure S7A,B). Consequently, we found that both S97A and S97D IRF3 mutant proteins interacted with DP96R at a similar level to WT IRF3, suggesting that DP96R does not interfere with the S97 of IRF3 (Figure 6A,B). Next, we conducted a competitive binding assay using IRF3 S97A and KPNA4 and added DP96R incrementally. Figure 6C shows that the interaction between IRF3 S97A and KPNA4 was inhibited by DP96R, suggesting that DP96R disrupts the KPNA-IRF3 interaction without interfering with the dephosphorylation of IRF3 S97.

To examine the IRF3-KPNA and IRF3-DP96R interactions at the amino acid level, we performed several immunoprecipitation assays using two mutant IRF3 plasmids. Previous studies suggested that the basic amino acid clusters K77/R78 and R86/K87 within IRF3, which occupy the minor and major binding sites within IRF3, respectively, are important for the nuclear translocation of IRF3 and may interact with KPNA [39,53]. The NLS region of IRF3, which contains these amino acid clusters, is highly conserved in most mammalian species (Figure S7C). Armed with this information, we performed a binding assay using K77N/R78G and R86L/K87Q double-mutant IRF3 plasmids, along with Flag-KPNA4 and Strep-DP96R. First, we checked the IRF3-KPNA4 and IRF3-DP96R interactions using the K77N/R78G IRF3 double-mutant plasmid. The mutant IRF3 protein still interacted with KPNA4 and DP96R (Figure 6D,E), indicating that DP96R does not interact with the KPNA4 minor binding site within IRF3. Second, we performed a similar interaction analysis using the R86L/K87Q double-mutant IRF3 plasmid. By contrast, we found that the mutant IRF3 protein interacted neither with KPNA4 (Figure 6F) nor with DP96R (Figure 6G), suggesting that DP96R targets the KPNA major interacting site within IRF3 exclusively to inhibit the IRF3-KPNA interaction. 

IRF7, which belongs to the IRF family of proteins, is activated by the cytosolic DNA-sensing pathway to generate IFNs. To test whether the interaction between DP96R and IRF3 is specific, we investigated the interaction between DP96R and IRF7. As shown in Figure S7D, IRF7 did not precipitate DP96R, suggesting that the interaction between endogenous IRF3 and DP96R is unique. Finally, we compared the NLS sequences of IRF3 and IRF7 and discovered that the KPNA-binding amino acids in IRF3 differ from those in IRF7 (Figure S7E). These findings strongly suggest that DP96R interacts with IRF3 via the IRF3 major binding site of KPNA, which then inhibits the IRF3-KPNA interaction and impairs the nuclear translocation of IRF3.

### 2.7. DP96R Is an Early Transcribed Protein Involved in the Antagonism of IFN and ISG Transcription

**Figure 7:**
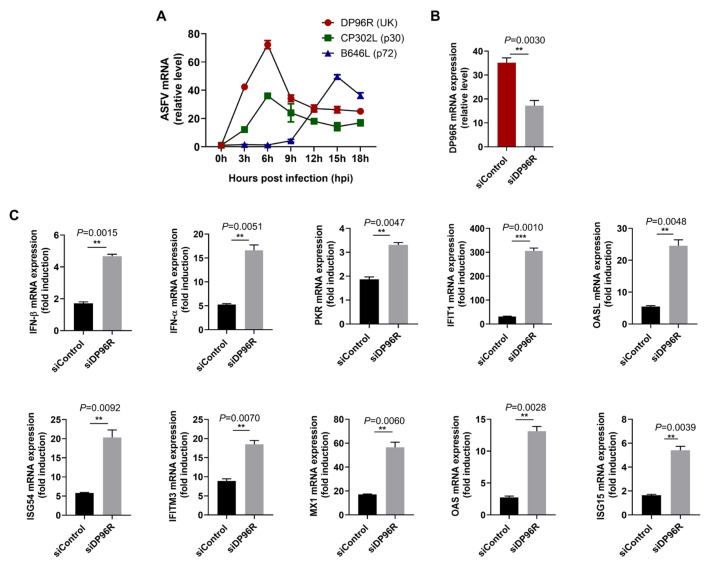
DP96R protein is transcribed early, inhibiting the transcription of IFNs and ISGs. Primary PAMs were infected with ASFV at 0.5 MOI and harvested at indicated time points. DP96R, CP302L, and B646L transcription expression levels during ASFV infection were determined by qRT-PCR. The p30 and p72 are shown as indicators of early and late genes, respectively (**A**). Primary PAMs in 24-well plates were transfected with the siRNAs against the DP96R or control siRNA for 6 h and then infected with the ASFV at an MOI of 0.5. The transcription levels of the indicated genes in the ASFV-infected primary PAMs were examined at 12 hpi (**B**,**C**). Data represents two independent experiments, each with similar results, and all the values are expressed as mean ± SD of two biological replicates. Student’s *t*-test: **, *p* < 0.01; ***, *p* < 0.001.

To assess the transcription kinetics of DP96R, total RNA was isolated from primary porcine alveolar macrophages (primary PAMs) infected with an ASFV (Korea/wild boar/Hwacheon/2020-2287) strain at a MOI of 0.5. The mRNA levels of the viral gene, relative to the cellular beta-actin (β-actin), were determined as outlined in previous protocols [58]. As depicted in Figure 7A, the transcription pattern of DP96R closely resembled that of CP302L, an early-transcribed gene, but differed from that of B646L, a late-transcribed gene. This observation supports the conclusion that DP96R is an early-transcribed gene of ASFV. To investigate the immunomodulatory functions of DP96R during ASFV infection, we knocked down DP96R mRNA that was induced by ASFV infection using DP96R-specific siRNA (siDP96R) in primary PAMs. Subsequently, these cells were infected with ASFV at a MOI of 0.5 for 12 h. DP96R mRNA was knocked down with almost 51% efficiency at 12 hpi (Figure 7B). In accordance with that, upon DP96R knock-down in ASFV-infected primary PAMs, the transcriptions of IFNs and ISGs were upregulated (Figure 7C). These data demonstrate that DP96R exerts inhibitory effects on IFN transcription, leading to reduced ISGs and thus inhibiting viral replication.

### 2.8. The Central Region of DP96R Regulates Immune Evasion

The results described so far suggest that ASFV DP96R inhibits the nuclear translocation of IRF3. Next, we aimed to pinpoint the precise region within DP96R that is responsible for inhibiting IFNs, cytokines, and chemokines. To do this, we created two plasmids that express truncated mutants of DP96R spanning aa 1–29 (D1) and 30–96 (D2) (Figure S8A); the construction of these mutants is described elsewhere [59]. Moreover, we further narrowed down DP96R, generating 30–60 (D3) and 61–96 (D4) fragments. Subsequently, we conducted IFN-β luciferase assays, mediated by TBK1 and IRF3-5D, and IRF3 interaction assays using truncated mutants of ASFV DP96R. The luciferase assays indicated that the D3 fragment of DP96R led to a decrease in luciferase activity, whereas the D1 and D4 fragments did not exhibit such an effect (Figure S8B,C). Next, we examined the interaction between DP96R fragments and IRF3. We found that the DP96R-D3 fragment is vital for this interaction (Figure S8D,E). Taken together, our findings suggest that the central region of DP96R, specifically amino acids 30–60, plays a pivotal role in suppressing the production of IFNs, pro-inflammatory cytokines, and chemokines by interacting with IRF3.

## 3. Discussion

Immune evasion mechanisms are important virulence factors. ASFV uses various immune evasion strategies to inhibit the production of IFNs and pro-inflammatory cytokines. These strategies involve manipulating and controlling multiple components within the IFN and NF-κB signaling pathways. Prior studies suggest that specific ASFV genes suppress host IFN or pro-inflammatory cytokine responses by targeting elements within the cGAS-STING-IRF3 and IKK-NF-κB signaling pathways. A recent study examined the idea that ASF viruses lacking these specific genes show reduced virulence, potentially serving as the basis for developing live attenuated vaccine strains [60]. The recombinant ASFV virus Benin ΔMGF, which lacks MGF family genes, shows impaired inhibition of IFN signaling, leading to reduced pathogenicity and protection of pigs against a subsequent challenge [61]. Infection with ASFV ΔMGF505-7R virus, which lacks the *MGF505-7R* gene, resulting in the induction of type I IFN production and NF-κB activation, also led to increased secretion of IFN-β in porcine serum and a reduction in viral virulence [6,23]. Additionally, ASFV CD2v, which encodes EP402R, and ASFV I267L regulate type I IFN signaling. Both ASFV ΔCD2v and ASFV ΔI267L show attenuated virulence in pigs [62,63]. Hence, investigating ASFV genes that are involved in evading IFN, or inflammatory responses may serve as a foundation for developing live attenuated ASFV vaccines.

ASFV *DP96R* encodes a protein that was previously demonstrated to be an inhibitor of cGAS-STING signaling [59], is transcribed towards the right end of the genome and does not exhibit significant similarity to other known genes in existing databases. Analysis of the *DP96R* gene sequence of various pathogenic ASFVs from Europe, the Caribbean, and Africa shows that this gene is remarkably conserved among pathogenic isolates, including those from ticks and pigs. In the late 1990s, a study demonstrated that the ASFV ΔDP96R virus lacking DP96R was not virulent in inoculated pigs and protected against challenge with homologous ASFV-E70 [35]. Therefore, a double-gene-deleted ASFV-G-Δ9GL/ΔDP96R virus [36], a seven-gene-deleted ASFV-HLJ/-18-7GD (including ASFV *UK*) virus [38], and a double-gene-deleted ASFV-SY18-∆CD2v/ΔDP96R virus [37] were constructed and showed attenuated virulence and protected immunized pigs from challenge with parental ASFV. Although the relationship between DP96R and ASFV virulence has been studied in vivo, the molecular mechanisms, including immune invasion related to DP96R, remain unknown.

Here, we identified a novel molecular mechanism by which ASFV DP96R inhibits IRF3-mediated antiviral immune responses. First, DP96R interacts with a crucial karyopherin (KPNA) binding site within IRF3. Second, in vitro mutagenesis of IRF3 showed that DP96R prevents interaction between activated IRF3 and KPNA and consequently impedes the translocation of IRF3 to the nucleus. Third, overexpression of DP96R boosts the replication of both DNA and RNA viruses by impeding the cGAS-STING signaling pathway and subsequent transcription of antiviral genes. Fourth, DP96R knock-down using DP96R-specific siRNA resulted in higher IFNs and ISG transcription during ASFV infection.

The IRF family in mammals includes IRF1, IRF2, IRF3, IRF4, IRF5, IRF6, IRF7, IRF8, and IRF9 [41,64,65]. Indeed, among IRF family members, IRF3 is a key transcription factor, crucial for initiating the production of type I IFNs. Expressing numerous genes that are involved in innate immune responses is essential and highlights their significance in antiviral defense mechanisms and overall immune regulation [66]. Upon infection by RNA or DNA viruses, activated TBK1 and IKKε/i phosphorylate IRF3 at specific serine residues [67,68]; and IRF3 then undergoes nuclear translocation to initiate the transcription of type I IFN by binding to specific regulatory elements known as IFN-stimulated response elements (ISREs). This process is crucial for activating the innate immune response against viral infections [69].

IRF3 comprises three key domains: AIE for phosphorylation, IAD for dimerization, and an NLS-associated DBD for nuclear translocation and binding to promoter DNA. The DBD and AIE of IRF3, but not the IAD, are highly conserved in humans and pigs. Given the central role of IRF3 in virus-induced innate immunity, many RNA or DNA viruses, including ASFV, have developed mechanisms to avoid or counteract IRF3-mediated responses within host cells [18,70]. Indeed, studies demonstrate that several viral proteins interact specifically with IRF3, which effectively hinders its nuclear translocation. For instance, the V protein of simian virus 5 [71]; the NS1 protein of influenza B virus [72]; the L protein of Theiler’s murine encephalomyelitis virus [73]; the ICP0 protein of HSV-1 [74]; and the ORF6 [75], NSP12 [76], and NSP5 [77] proteins of SARS-CoV-2 all inhibit the nuclear translocation of IRF3.

Reports suggest that the inhibition of the nuclear translocation of phosphorylated IRF3 in the virulent isolate ASFV Armenia/07 is greater than that of IRF3 in the non-virulent ASFV isolate NH/P68 [21], suggesting that there are specific ASFV proteins that impair the type I IFN signaling cascade by inhibiting the translocation of IRF3 to the nucleus. A recent study showed that MGF 505-7R of ASFV-HLJ/18 associates with IRF3 to inhibit the nuclear translocation of IRF3, although the mechanism is unclear [6]. Here, we show that ASFV DP96R interacts with a critical binding site in IRF3, which is essential for the interaction with KPNA. This interaction between DP96R and IRF3 effectively hinders the translocation of IRF3 to the nucleus by disrupting the IRF3-KPNA interaction. KPNA molecules play a pivotal role in the nuclear translocation of IRF3 by acting as an adaptor protein that links NLS-containing proteins (e.g., IRF3, IRF7, P65, and STAT1) with KPNB [78]. The KPNA family comprises KPNA1–KPNA6. Previously, Zeng Cai et al. showed that IRF3 only interacts with KPNA2, although this result may be due to the disproportionate expression of KPNA molecules in the experiments [56]. In the current study, we show that IRF3 interacts with KPNA1, KPNA2, KPNA3, and KPNA4 (Figure 5A,B) and that DP96R impedes interaction between IRF3 and all of these molecules (Figure 5C–H).

Shun Li et al. demonstrated that S97 of IRF3 must be dephosphorylated for nuclear translocation of IRF3 [57], and Zeng Cai et al. showed that KPNA2 interacts with IRF3 when S97 is dephosphorylated [56]. Therefore, we asked whether DP96R inhibits the dephosphorylation of IRF3 S97 or blocks the KPNA-S97 dephosphorylated IRF3 interaction. When we used an S97A mutant of IRF3, DP96R was not involved in the dephosphorylation of IRF3 S97, and the interaction between dephosphorylated IRF3 with KPNA4 was still disrupted by ASFV DP96R, suggesting that DP96R blocks the KPNA-S97-dephosphorylated IRF3 (Figure 6C).

The IRF3 protein possesses a bipartite NLS that is defined by two clusters of basic amino acids: one formed by lysine 77 (K77) and arginine 78 (R78), located at the minor binding site, and the other comprising arginine 86 (R86) and lysine 87 (K87), located at the major binding site of KPNA. Notably, prior to our study, there was no evidence of an interaction between these components [39]. Here, we demonstrate that R86 and K87 of IRF3 are necessary for the interaction with KPNA, whereas K77 and R78 of IRF3 are not (Figure 6D,F). Specifically, DP96R also targets R86 and K87 of IRF3 (Figure 6G). These results suggest that DP96R competes with KPNA molecules for binding to IRF3 without affecting the dephosphorylation of S97.

Previously, Wang et al. also described a mechanism of cGAS-STING pathway inhibition by DP96R degrading TBK1 [59]. However, we did not observe evidence of endogenous or overexpressed TBK1 degradation by DP96R in a dose-dependent manner in our condition. Moreover, our luciferase data showed that DP96R dose-dependently reduces IRF3 and IRF3-5D that are mediated IFN-β luciferase activity. During our study, we depended on the dosage-dependent effects of DP96R, which could potentially be a determining factor in the varied outcomes obtained. In summary, we report a novel molecular mechanism by which DP96R acts as a negative modulator of type I IFNs and pro-inflammatory responses by impairing the nuclear translocation of IRF3. These data increase our understanding of the connection between DP96R and the virulence of ASFV and reinforce the idea that DP96R represents a crucial target for the development of live attenuated vaccines.

## 4. Materials and Methods

### 4.1. Cells and Antibodies

HEK293T cells (ATCC^®^ CRL11268™), 293-Dual™ hSTING-A162 cells (Invivogen, San Diego, CA, USA, 293d-a162), PK-15 cells (ATCC^®^ CCL-33), A549 cells (ATCC CCL-185), Vero cells (ATCC^®^ CCL-81™), and MA104 cells were cultured in Dulbecco’s Modified Eagle Medium (DMEM) (Cytiva, Marlborough, MA, USA) media and PAMs (ATCC^®^ CRL2843™) in Roswell Park Memorial Institute Medium (RPMI) (Cytiva) media. For the culture of PIBs, RPMI media were utilized, supplemented with 5% Glutamax (Gibco, Waltham, MA, USA) along with 300 µL of normacin (Invivogen). Each cell type was provided with 10% fetal bovine serum (Gibco) and 1% antibiotic/antimycotic (AA) (Gibco). Subsequently, they were placed in a humidified incubator with 5% CO_2_ at 37 °C. Primary PAM (Optipharm Inc., Cheongju, Republic of Korea) was cultured in 10% fetal bovine serum (FBS) (Gibco™) and 1% Penicillin-Streptomycin (Gibco™)-added RPMI-1640 medium (Hyclone™, Cytiva) in an incubator at 37 °C and 5% CO_2_ atmosphere. The antibodies employed for both immunoblot and immunoprecipitation analyses are as follows: Flag-mouse (Cell Signaling, Danvers, MA, USA, 8146), Flag-Rabbit (Sigma, St. Louis, MO, USA, F7425), GST (Santa Cruz, sc-138), Strep (QIAGEN, Venlo, The Netherlands, 34850), IRF3 (Cell Signaling 4302s), phospho IRF3 (Ser396) (Cell Signaling, 4947), IKKα (Santa Cruz, Dallas, TX, USA, sc-7606), phospho IKKα/β (Cell Signaling, 2697S), p65 (Cell Signaling, 4764S), phospho p65 (Cell Signaling, 3031S), TBK1 (Cell Signaling, 3504S), phospho TBK1 (Cell Signaling, 5483S), phospho IҡBα (Cell Signaling, 2859S), IҡBα (Cell Signaling, 9242S), STAT1 (Cell Signaling, 9172S), pSTAT1 (Cell Signaling, 9167S), KPNA2 (Santa Cruz, sc-55538), KPNA4 (Santa Cruz, 390535), α-tubulin (Santa Cruz, sc-8035), Lamin B1(Cell Signaling, 12586), and β-actin (Santa Cruz, SC 47778). For the immunofluorescence assay, cy3-conjugated donkey mouse IgG (The Jackson Laboratory, Bar Harbor, ME, USA, 715-165-150) and Alexa 488 goat rabbit IgG (Invivogen, A11034) were used. These antibodies were utilized following the manufacturer’s provided instructions.

### 4.2. Porcine Bone Marrow-Derived Macrophage Isolation

Cells derived from porcine bone marrow were isolated following the subsequent protocols [79,80,81]: Five ribs from the rear portion were extracted from each side of the animal, and the bone surfaces were cleansed with alcohol. Both ends were incised, and the bone marrow was flushed from both sides using a 20 mL syringe and an 18 G needle, with RPMI 1640 (Cytiva) supplemented with 5 mM EDTA (Gibco) to prevent clotting. The cells were filtered, subjected to centrifugation, and then suspended in red blood cell lysis buffer (ACK lysing buffer, Gibco) for 2 min. After the cells were lysed, they underwent centrifugation, and the resulting pellet was washed first with PBS and then with RPMI 1640. Lastly, the bone marrow cells were suspended in a freezing medium consisting of 90% heat-inactivated FBS and 10% DMSO. They were then frozen overnight in a “Mr. Frosty” isopropanol box (Nalgene, Rochester, NY, USA) at −80 °C, enabling gradual temperature reduction for controlled freezing. On the subsequent day, the cells were transferred to a freezer set at a temperature of −196 °C for extended, long-term storage. To retrieve the cells from the freezer, they were rapidly thawed in a 37 °C water bath and then cautiously diluted by gradually adding complete medium drop by drop over 2–3 min. This gradual dilution was performed to prevent any abrupt changes in osmolarity due to DMSO. The cells were slowly diluted by adding 40 mL of warm PBS drop by drop over a 2–3-min period, preventing the sudden dilution shock from DMSO. Following the DMSO removal through washing, the cells were cultured in a complete medium consisting of RPMI 1640, 10% heat-inactivated FBS (Gibco), AA (Gibco), and GlutaMAX-I supplement (Gibco). Porcine BMDMs were generated by culturing bone marrow cells on sterile 100 mm^2^ petri dishes for 5–7 days in the presence of pGM-CSF-1 (20 ng/mL; R&D Systems, Minneapolis, MN, USA, 711-pg-010). The obtained macrophages were detached by forcefully splashing them with a medium using a syringe and an 18 G needle. Afterward, they were washed, counted, and then seeded into tissue culture plates at a concentration of 1 million cells per milliliter (10^6^ cells/mL) in a medium containing pGM-CSF-1.

### 4.3. Porcine Bone Marrow-Derived Macrophage Immortalization

Before the lentiviral transduction, a G418 dose selection assay was performed, and the minimum G418 concentration for the onset of pBMDM cell death was determined. First, pBMDMs were allowed to differentiate for 5–7 days. Then, the pBMDMs were split into a 24-well cell culture plate. For G410, 100µg/mL to 400 µg/mL doses were tested, and cell death was shown at 400 µg/mL. Subsequently, primary porcine BMDMs were divided and placed into individual wells of a 24-well cell culture plate. Each well contained 0.5 mL of RPMI1640 supplemented with 10% FBS, GlutaMax-I, and AA, with a cell density of 1 million cells (1 × 10^6^ cells) per well. The plates were then incubated at 37 °C in a 5% CO_2_ environment overnight.

At the time of transduction, cell confluence was 50–70%. Preformed lentiviral particles expressing SV40 large T antigen under the CMV promoter and containing neomycin markers were purchased (AMSBIO, Abingdon, UK). The calculated virus particle and polybrene levels were 5 µL and 0.6 µg/µL, respectively. A determined amount of retrovirus was added into 1.5 mL microcentrifuge tubes, bringing the volume up to 0.5 mL using complete media. Polybrene was introduced into the mixture to achieve a final concentration of 8 µg/mL. The medium was removed from the cell culture plate, and 500 µL of the prepared mixture of complete medium, including lentivirus and polybrene, was added without disturbing the cells. The cells were cultured with the virus for a period of 48 to 72 h in a 37 °C incubator under 5% CO_2_ conditions.

Three days after transduction, cells were treated with complete media containing 800 µg/mL G418 to select resistant cells. The integration of hTERT and SV40LT was verified using PCR. For the template for PCR, the genomic DNA was isolated using an RNA/DNA mini kit. PCR primers for hTERT were as follows: forward primer- 5′-GCCGAGACCAAGCACTTCCTCTACT-3′, reverse primer-5′-GCAACTTGCTCCAGACACTCTTCCG-3′ and SV40LT; forward primer- 5′-GATGGCTGGAGTTGCTTGGCTACAC-3′, reverse primer-5′GCCTGAAATGAGCCTTG GGACTGTG-3′. The PCR protocol included an initial denaturation cycle of 5 min at 95 °C, followed by 35 cycles consisting of 30 s at 94 °C, 30 s at 63 °C, and 45 s at 72 °C. Finally, there was a concluding extension step of 5 min at 72 °C.

### 4.4. Continuous ASFV DP96R Protein-Expressing Cell Generation

DP96R continuously expressing PAMs, PIBs, and MA104 cells were generated by transient transfection of the DP96R-pIRES-Flag plasmid with Lipofectamine 2000 (Invitrogen, Carlsbad, CA, USA). Positive colonies were selected in 10% FBS-containing DMEM treated with 2 µg/mL, 0.3 µg/mL, or 4 µg/mL puromycin (Thermo Fisher Scientific, Waltham, MA, USA) for each cell line for at least two weeks. The presence of DP96R protein in the cells was validated through immunoblotting using anti-Flag antibodies.

### 4.5. Plasmids

The complete form of the ASFV DP96R protein-expressing sequence (GenBank, Bethesda, MD, USA: FR682468.1) was cloned into Flag-tagged pIRES, Strep-tagged pEXPR, and GST-tagged pEGB vectors. Truncated DP96R gene fragments were cloned into a pEGB vector with a GST tag. To generate IRF3 different constructs, IRF3 was amplified from template DNA using PCR and cloned into pIRES-Flag, pEXPR-Strep, or pEBG-GST vectors. IRF3 domains were subcloned into the pEGB vector. The mutation cloning kit (Thermo Fisher, 00940669) generated the IRF3 point mutated sequences. KPNA (Importin α) 1 to 6 were cloned into a pIRES vector with a Flag tag. Generation procedures of the IFN-β and NF-κB promoter and luciferase reporter plasmids have been described elsewhere [82]. cGAS 3× Flag-tagged plasmid was kindly donated by Dr Jae U. Jung (Lerner Research Institute, 9500 Euclid Avenue, Cleveland, OH, USA). Flag-tagged STING, TBK1, IKKε, IRF3, P65, and V5-tagged TBK1 were generated by amplifying template DNA and cloning it into the pIRES vector. The integrity of all sequences was verified by sequencing analysis. Plasmids utilized in the studies encode human proteins.

### 4.6. Virus Infection and Plasmid Transfection

ADV-GFP was amplified in PK-15 cells, while HSV-GFP and VACV-GFP were amplified in Vero cells and quantified using a plaque assay. The Sendai virus Cantell strain, GFP-tagged H_1_N_1_ virus-PR8 strain (PR8-GFP), and NDV-GFP were amplified in specific pathogen-free (SPF) eggs. Before virus infection, the culture medium was replaced with DMEM containing 1% FBS, and the virus was introduced into the target cells at a specific multiplicity of infection (MOI). After a 2 h incubation at 37 °C, the extracellular viruses were eliminated, and the medium was substituted with DMEM containing 10% FBS. At the specified time points, the cells were detached from the culture plates along with the supernatants, and the mixture was subjected to centrifugation at 3000 rpm for 3 min. The supernatant of each sample was separated from the cell pellet for ELISA. The cell pellet was reconstituted in 300 µL of phosphate-buffered saline (PBS), and the fluorescence of each sample was assessed using a fluorometer, specifically, the Glomax detection system from Promega. The plasmids were introduced into PK-15, PAMs, PIBs, and MA104 cells using Lipofectamine 2000 (Invitrogen), and for HEK293T and 293-Dual™ hSTING-A162 cells, Polyethylenimine (PEI; Polyscience Inc., Warrington, PA, USA, 23966) was employed for transfection. The procedures followed the manufacturer’s protocol.

### 4.7. Virus Titration

Virus-infected cells and cell culture supernatants were collected at the indicated time points, and viruses were titrated by plaque assay using A549 cells for ADV-GFP and VACV-GFP and *Ceropithecus aethiops* epithelial kidney (Vero) cells for HSV-GFP, PR8-GFP, and NDV-GFP. A549 and Vero cells were placed into 12-well plates and allowed to incubate for a duration of 12 h. Subsequently, the cells were exposed to supernatants containing the virus that had been serially diluted, using 1% DMEM, for a period of 2 h. After the incubation period, the inoculums were discarded and substituted with DMEM containing 0.1% agarose (from Sigma-Aldrich). The plates were incubated for 36 h at 37 °C and subsequently examined for plaque formation under a magnification of 200×. Determining virus titers was performed by employing the number of plaque-forming units (PFUs) and the dilution factor in the calculations.

### 4.8. DP96R Transcription Assay

In the ASFV experiment, primary porcine alveolar macrophages (primary PAMs) were exposed to a 0.5 multiplicity of infection (MOI) of ASFV (Korea/wild boar/Hwacheon/2020-2287). Cell pellets were collected at 0, 3, 6, 9, 12, 15, and 18 hpi. Subsequently, RNA extraction was carried out, and qRT-PCR analysis was performed using DP96R primers (Table S1). All experiments dealing with ASFV were conducted in accordance with the Standard Operating Procedure (SOP) in the biosafety level 3 (BSL-3) laboratory of the NIWDC in Korea.

### 4.9. RNA Interference Experiments

The specific small interfering RNA (siRNA) for targeting ASFV DP96R was designed and synthesized by Bioneer (Daejeon, Republic of Korea). Primary PAMs (cell number 1 × 10^6^ in 24-well plates) were transfected with negative control (siControl) or DP96R (siDP96R) siRNA by RNAimax (Invivogen). The target sequence of DP96R was 256- GGAUCCCUAAUGCGCUCCA-274. Nontargeting siRNA was used as a negative control (UUCUCCGAACGUGUCACGU). At 6 hpt, the cells were left uninfected or infected with ASFV at a multiplicity of infection (MOI) of 0.5. The mRNA expression levels of the target gene, IFNs, and ISG mRNA were detected by qRT-PCR. All experiments dealing with ASFV were conducted in accordance with the Standard Operating Procedure (SOP) in the biosafety level 3 (BSL-3) laboratory of the NIWDC in Republic of Korea.

### 4.10. ELISA

ELISA was conducted to identify the presence of secreted interferons, pro-inflammatory cytokines, and chemokines within the cell culture supernatants. Human IL-6 (BD OptEIA, Franklin Lakes, NJ, USA, 5552220), Human IFN-β (CUSABIO, CSB-E09889h), porcine IL-6 (R&D Systems, p6000B), and porcine IFN-β (CUSABIO, CSB-E09890p), were used for analysis, and the procedures followed the manufacturer’s provided protocols.

### 4.11. Quantitative Real-Time PCR

DP96R pIRES or pIRES stably expressing PAMs were cultivated in 6-well tissue culture plates, with a seeding density of 1 × 10^6^ cells per well, and maintained at a temperature of 37 °C. The cells were infected with ADV-GFP and HSV-GFP at a multiplicity of infection (MOI) of 1 and then collected at 0, 12, and 24 hpi. The total RNA from the cells was extracted using the Machery Nagel Nucleospin RNA kit (790955.250), and subsequently, cDNA was synthesized using reverse transcriptase (Toyobo, Osaka, Japan). The varying levels of cDNA were quantified through real-time polymerase chain reaction (RT-PCR) employing the Smart Gene SYBR Green Q-PCR master mix (SG. SYBR.500) kit, following the manufacturer’s provided instructions. The primer sequences utilized in the quantitative polymerase chain reaction (qPCR) are detailed in Table S1.

### 4.12. Immunoprecipitation

At 36 h post-transfection (hpt), the cells were collected, and whole-cell lysates (WCLs) were acquired following lysis using a lysis buffer containing protease inhibitor cocktail (PI), phosphatase inhibitor cocktail (Sigma), and radio-immunoprecipitation assay (RIPA) lysis buffer (comprising 50 mM Tris-HCl, 150 mM NaCl, 0.5% sodium deoxycholate, 1% IGEPAL, 1 mM NaF, and 1 mM Na3VO4). The lysates were then sonicated using a sonicator from Sonics. The whole-cell lysate (WCL) was initially subjected to a pre-clearing step by incubation with Sepharose 6B (GE Healthcare Life Science, Little Chalfont, UK) at 4 °C with rotation for 2 h. Following the pre-clearing step, for GST and Strep pull-down, the WCL was subjected to incubation with a 50% slurry of glutathione-conjugated Sepharose (GST) beads (Amersham Biosciences, Slough, UK) and Strep beads (IBA) for 12 h. In contrast, for antibody pull-down, the specified antibodies were incubated with WCL for 12 h, and protein A/G PLUS-Agarose beads (Santa Cruz, H0422) were employed for incubation, lasting 4 h. The immunoprecipitated beads were gathered following centrifugation and subjected to washing with lysis buffer using various washing conditions. Subsequently, the interaction between the relevant proteins was assessed through immunoblot analysis.

### 4.13. Immunoblot Analysis

The cell lysates prepared with immunoprecipitated beads were separated via SDS-PAGE and then transferred to a PVDF membrane using a Trans-Blot^®^ semi-dry transfer cell from Bio-Rad located in Seoul, Republic of Korea. Following the transfer, the membrane was blocked for a duration of 1 h using 5% bovine serum albumin (Georgiachem, Norcross, GA, USA, BS1005) and subsequently incubated overnight at 4 °C with the primary antibody. Subsequently, the membranes were subjected to washing with either TBST or PBST, and then, the membrane was incubated with a horseradish peroxidase-conjugated (HRP) secondary antibody (GeneTex, Irvine, CA, USA, GTX213111-01) for a period of 2 h at room temperature. The membrane underwent three rounds of washing with TBST or PBST. Lastly, the reaction was visualized using an enhanced chemiluminescence detection system (ECL) (GE Healthcare, located in Little Chalfont, UK) and with Amersham ImageQuant 800 (Cytiva).

### 4.14. Luciferase Reporter Assay

HEK293T cells and 293-Dual™ hSTING-A162 cells were cultured in 12-well tissue culture plates (3.5 × 10^5^ cells/well) and incubated at 37 °C. The human STING-overexpressing HEK293T cell line, 293-dual hSTING-A162, was used to examine the IFN-β luciferase activity induced by poly(dA:dT), cGAS and 2′3′cGAMP, while HEK293T cells were used to test the STING-, TBK1-, IKKε-, IRF3- and IRF3-5D-induced IFN-β luciferase reporter assay. HEK293T cells were transfected with IFN-β luminescence, TK renilla-luciferase reporter plasmid (an internal control to normalize transfection efficiency). DP96R pIRES plasmid or control vector was dose-dependently transfected. poly(dA:dT) (InvivoGen) and 2′3′cGAMP ligands (InvivoGen) were transfected with Lipofectamine 2000 (Invitrogen) and Lipofectamine RNAi max (Invitrogen), respectively. At 24 h post-transfection, cells were washed with phosphate-buffered saline (PBS) and lysed with 1× Passive Lysis buffer (Promega, Madison, WI, USA) for 15 min. Following the manufacturer’s protocol, luciferase activity was measured using a dual-luciferase reporter assay system (Promega; E1980).

### 4.15. Nuclear Fractionation Assay

In a 6-well cell culture plate, 80–90% confluent DP96R protein stably expressing PAMs, infected with ADV-GFP 1MOI for 2 h, were harvested at specified time intervals. Then, nuclear and cytoplasmic extraction procedures were performed following the instructions provided by the manufacturer (Invent Biotechnologies Inc., Plymouth, MN, USA, SM-005). The concentration of cytosol or nuclear lysate was quantified using the Bradford assay (Bio-Rad, Hercules, CA, USA).

### 4.16. Immunofluorescence and Confocal Microscopy

PK-15 cells were placed into an eight-well chamber slide manufactured by Ibidi. Harvested cells were fixed by incubating them with 4% paraformaldehyde for a duration of 20 min at room temperature. The fixed cells were rinsed with 1× PBS and then made permeable by treating them with absolute methanol. This was followed by an incubation period of 20 min at −20 °C. The cells were rinsed with 1× PBS and subsequently blocked by exposing them to 2% BSA in 1× PBS for a duration of 1 h at room temperature. The primary antibodies were added and incubated at 4 °C overnight. Following this, the cells underwent three rounds of washing with 1× PBST and were then exposed to an appropriate secondary antibody for a period of 1 h at room temperature. Subsequently, the cells were subjected to three additional washes with 1× PBST and stained with DAPI (4′,6-diamidino-2-phenylindole) for a duration of 10 min at room temperature. Images were captured using a LEICA DMi8 microscope and then analyzed with LAS-X software (version 3.7.1.21655). Pearson correlation coefficient of confocal images was calculated using Fiji Image J (version 1.54c); an open-source platform for biological image analysis.

### 4.17. Statistical Analysis

Graphs and all statistical analyses were performed using GraphPad Prism software version 6 for Windows. Data are presented as the means and standard deviations (SDs) representing at least two independent experiments. An unpaired Student’s *t*-test was performed to compare the control and treatment groups at each time point. *p* values of <0.05, <0.01, <0.001, or <0.0001 were considered significant.

## Data Availability

All the pertinent data can be found within the manuscript and its accompanying supporting information files. The authors confirm that all data supporting the findings of this study are available at figshare (https://figshare.com/s/89a1f90c6eab8dc7d4fd) (accessed on 27 November 2023).

## Supplementary Materials

The following supporting information can be downloaded at: https://www.mdpi.com/article/10.3390/ijms25042099/s1.
