# Supplementary material for: The African Swine Fever Virus Virulence Determinant DP96R Suppresses Type I IFN Production Targeting IRF3
Source: Int J Mol Sci. 2024 Feb 8;25(4):2099. doi: 10.3390/ijms25042099 (PMC10889005; doi:10.3390/ijms25042099)
Supplement: Supplementary file 1 [file ijms-25-02099-s001.zip › ijms-2824436-supplementary.pdf]

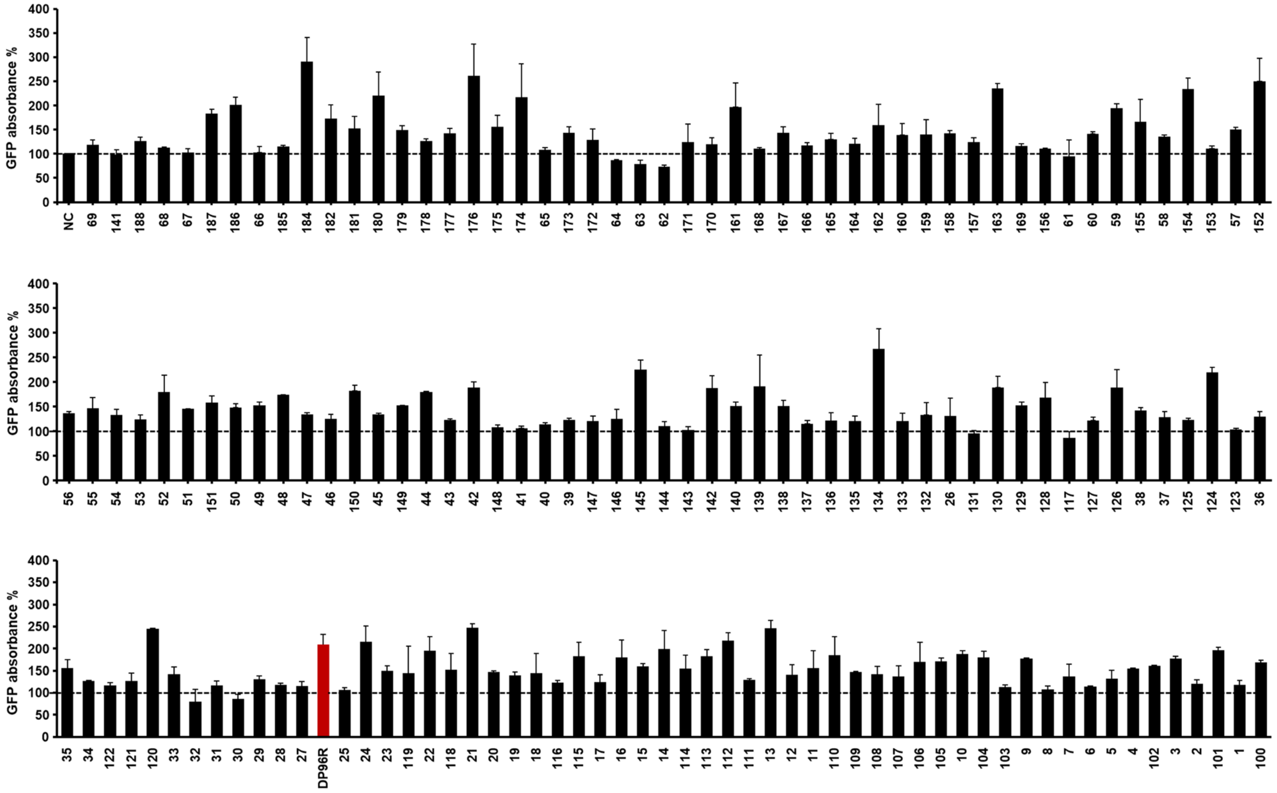

Figure S1. Adenovirus screening results of ASFV genes library. PK-15 cells were transiently transfected with indicated individual ASFV genes for 24 h. Virus-infected cells were harvested after 24 hpi, and the harvested cell pellets were subjected to quantify the replicated virus amount using a fluorescence modulator.

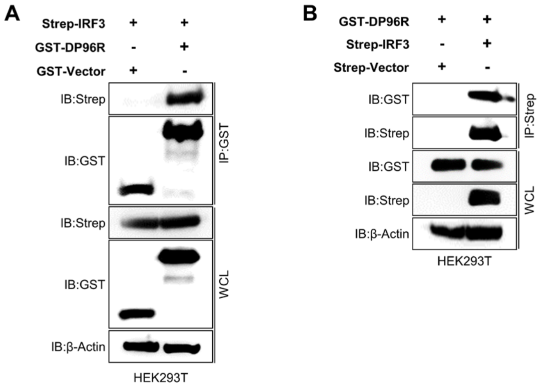

Figure S2. ASFV DP96R interacts with IRF3 to downregulate innate immune responses. (A) HEK293T cells were transfected with Strep-IRF3 and GST-DP96R and its control plasmids. Cell lysates were subjected to GST PD, followed by immunoblot with anti-Strep and -GST antibodies. (B) HEK293T cells were transfected with GST-DP96R and Strep-IRF3 and its control plasmids. Cell lysates were subjected to Strep IP and immunoblotting with anti-Strep and -GST antibodies. Data represent at least two independent experiments, each with similar results.

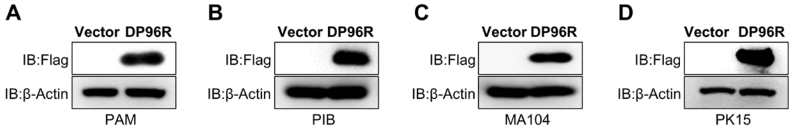

Figure S3. Detection of ASFV DP96R expressions by western blot using anti-Flag antibody. Overexpression of DP96R in (A) PAMs, (B) PIBs, (C) MA104, and (D) PK-15 cells. Data represent at least two independent experiments, each with similar results.

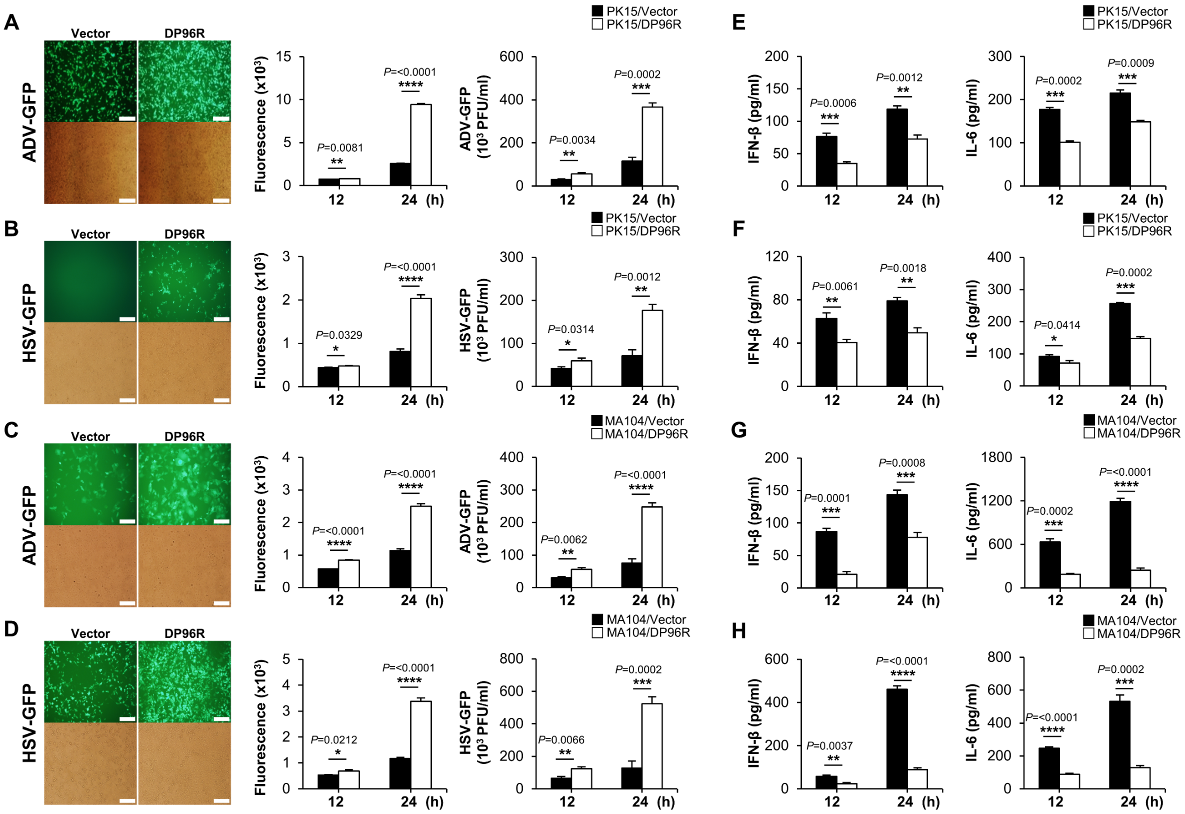

Figure S4. DP96R negatively regulates ADV-GFP and HSV-GFP-induced anti-viral innate immune responses. Flag-DP96R protein expressing (A and B) PK-15 cells (C and D) and MA104 with Flag-control cells were infected with ADV-GFP (1MOI) (A and C) and HSV-GFP (B and D). Viral replication was determined at 24 hpi by GFP expression levels by fluorescence microscopy and quantified at 12 hpi and 24 hpi by a fluorescence modulator. Virus titers of each sample were determined by plaque assay in A549 and Vero cells. Porcine IFN-β, IL-6, and Human IFN-β, IL-6 secretion in cell culture supernatant at 12 hpi and 24 hpi were determined by ELISA (E-H). Data represent at least two independent experiments, each with similar results, and the values are expressed as mean ±SD of three biological replicates. The scale bar represents 50 μM. Student's *t*-test: *, P < 0.05; **, P < 0.01; ***, P < 0.001; ****, P < 0.0001.

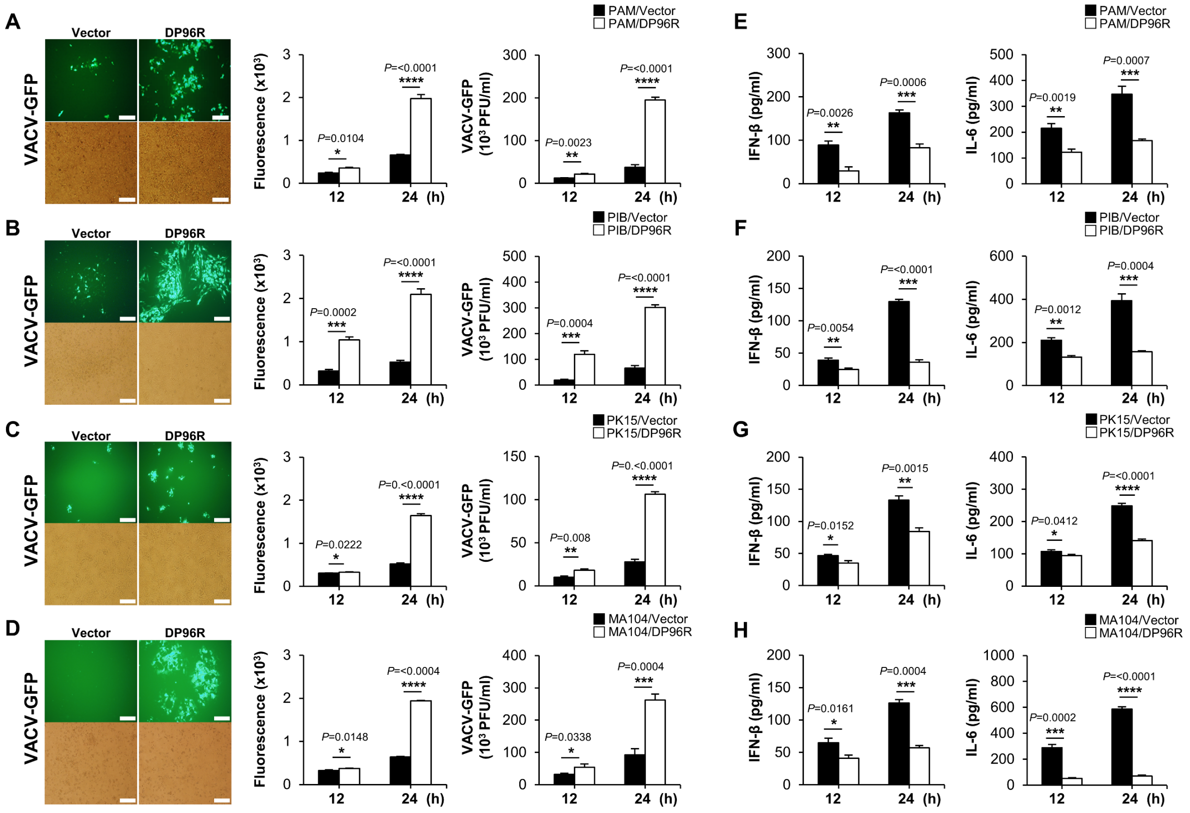

Figure S5. DP96R negatively regulates VACV-GFP-induced anti-viral innate immune responses. Flag-DP96R protein expressing PAMs (A), PIBs (B), PK-15 cells (C), and MA104 (D) with Flag-control cells were infected with VACV-GFP (1MOI). Viral replication was determined by fluorescence microscopy at 24 hpi by GFP expression levels and quantified at 12 hpi and 24 hpi by a fluorescence modulator. Virus titers of each sample were determined by plaque assay in Vero cells. Porcine IFN-β, IL-6, and Human IFN-β, IL-6 secretion in cell culture supernatant at 12 hpi and 24 hpi were determined by ELISA (E-H). Data represent at least two independent experiments, each with similar results, and the values are expressed as mean ±SD of three biological replicates. The scale bar represents 50 μM. Student's *t*-test: *, P < 0.05; **, P < 0.01; ***, P < 0.001; ****, P < 0.0001.

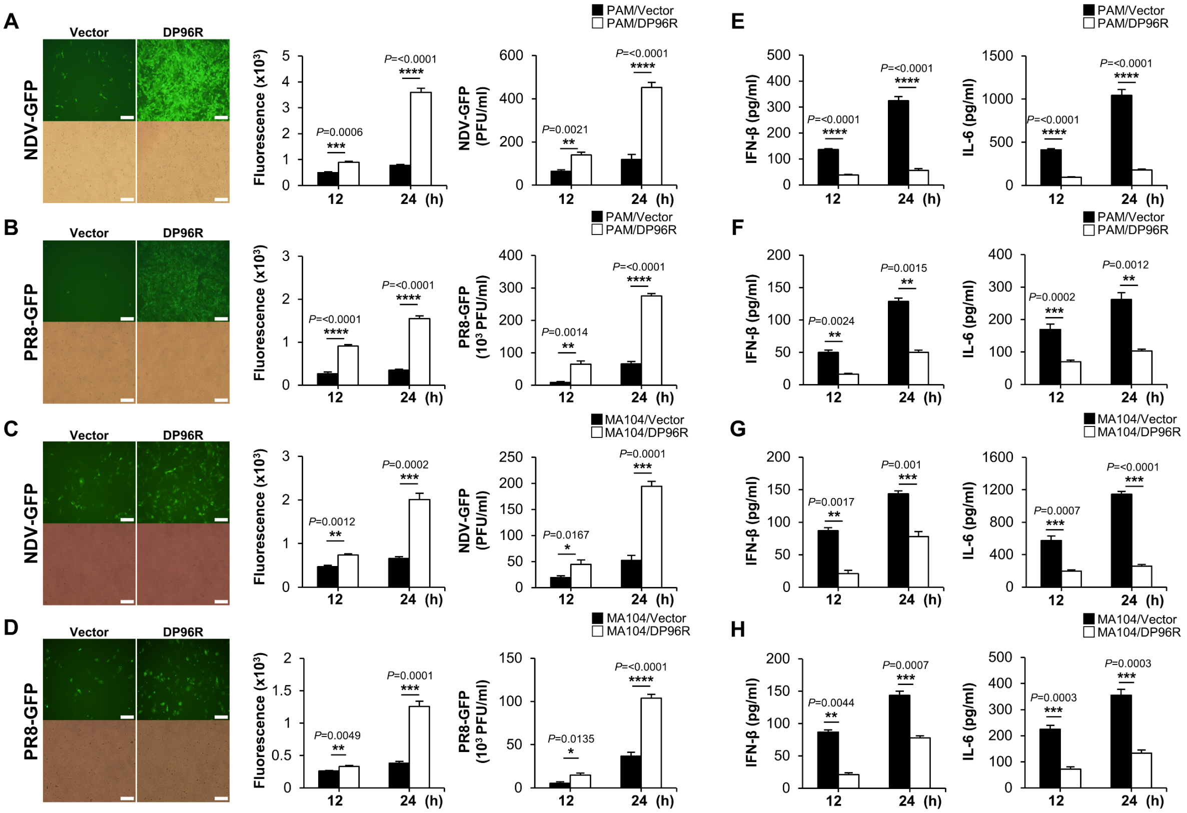

Figure S6. DP96R negatively regulates RNA virus-induced anti-viral innate immune responses. Flag-DP96R protein stably expressing (A and B) PAMs (C and D) and MA104 cells with Flag-control cells were infected with NDV-GFP (1MOI) (A and C) and PR8-GFP (B and D). Viral replication was determined at 24 hpi by GFP expression levels by fluorescence microscopy and quantified at 12 hpi and 24 hpi by a fluorescence modulator. Virus titers of each sample were determined by plaque assay in Vero cells. Porcine IFN-β, IL-6, and Human IFN-β, IL-6 secretion in cell culture supernatant at 12 hpi and 24 hpi were determined by ELISA (E-H). Data represent at least two independent experiments, each with similar results, and the values are expressed as mean ±SD of three biological replicates. The scale bar represents 50 μM. Student's *t*-test: *, P < 0.05; **, P < 0.01; ***, P < 0.001; ****, P < 0.0001.

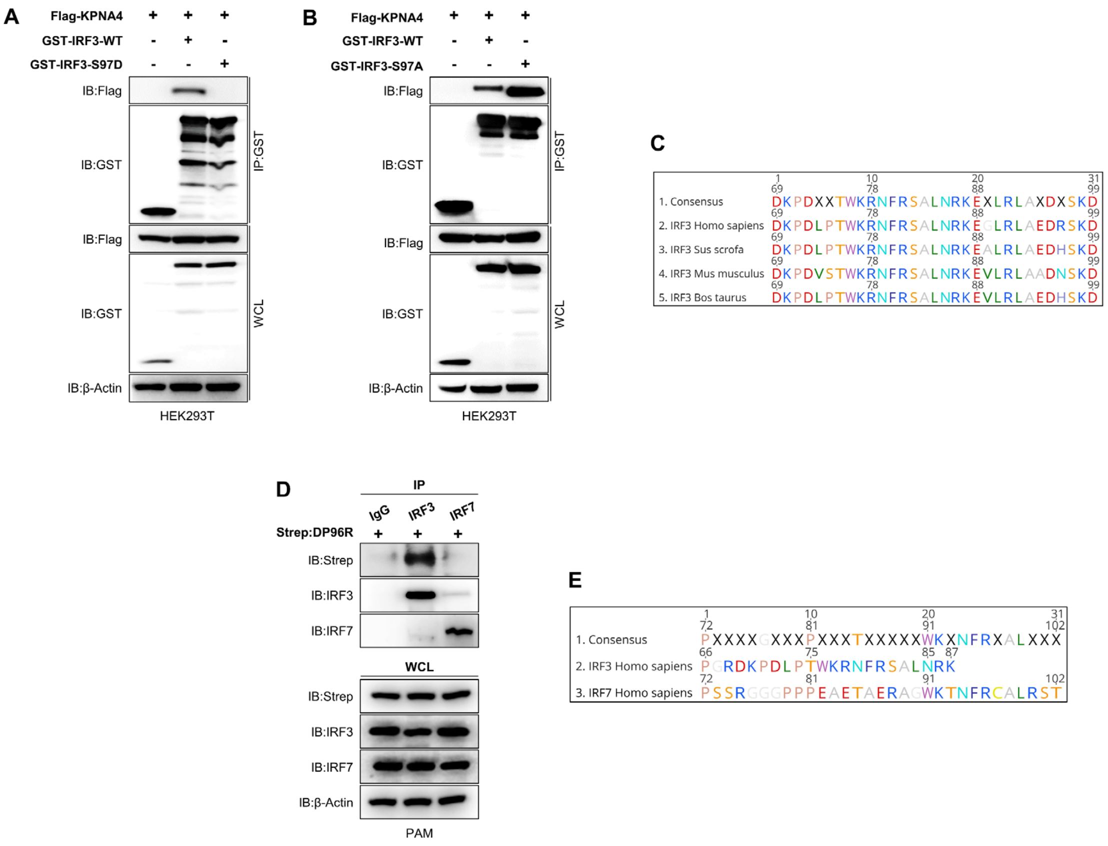

Figure S7. IRF3 S97 dephosphorylation augments IRF3-KPNA interaction, and DP96R does not interact with IRF7. HEK293T cells were transfected with GST-IRF3-WT, GST-IRF3 (S97D) (A), GST-IRF3 (S97A) (B) and its control plasmid along with Flag-KPNA4. Cell lysates were subjected to GST PD and immunoblotting with anti-Flag and -GST antibodies. (C) The conserved status of IRF3-NLS region in human, pig, mouse, and bovine (D) PAMs was transfected with Strep-DP96R plasmid. Cell lysates were subjected to IgG, IRF3, and IRF7 antibody IP and immunoblotted with anti-Strep, anti-IRF3, and -IRF7 antibodies. (E) Sequence alignment between human IRF3 (aa 66-87) and human IRF7 (aa 72-102). Data represent at least two independent experiments, each with similar results.

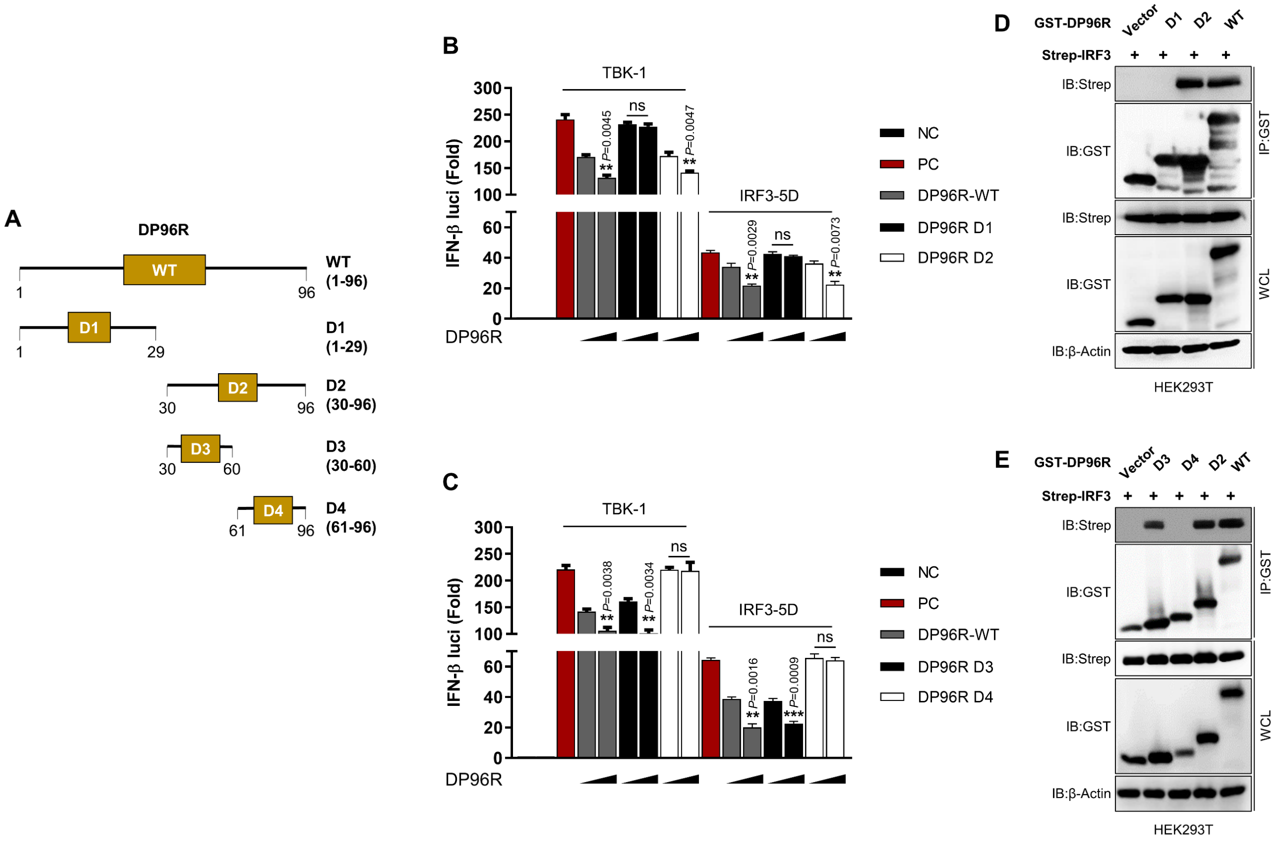

Figure S8. DP96R central region determines the pathogenicity. (A) ASFV DP96R truncation mutant map. (B) TBK1 and IRF3-5D mediated IFN-β luciferase assay was performed using GST-tagged full-length,aa 1-29, 30-60, 30-96, and 61-96 fragments of DP96R dose-dependently in HEK293T cells (B and C). GST-tagged full-length, aa 1-29, 30-60, 30-96, and 61-96 fragments of DP96R, its control vector, and Strep-IRF3 plasmids were co-transfected into HEK293T cells. Cell lysates were subjected to GST PD and immunoblotted with anti-Strep and -GST antibodies (D and E). Luciferase data represent three independent experiments, each with similar results, and all the values are expressed as mean ± SD of two biological replicates. All the immunoblot and confocal data represent at least two independent experiments, each with similar results. Student's *t*-test: **, P < 0.01; ***, P<0.001; ns, not significant.

Gene	Forward primer	Reverse primer
pIFN-β	AAATCGCTCTCTGATGTGT	TGCTCCTTTGTTGGTATCG
pIFN-γ	CCATTCAAAGGAGCATGGAT	ATCCATGCTCCTTTGAATGG
pIL-6	CACCGGTCTTGTGGAGTTTC	GTGGTGGCTTTGTCTGGATT
pIFIT1	CTGACTCACAGCAACCATG	CTTTCAGGTGTTTCACATAGG
pISG-15	AAATCGCTCTCCTGATGTGT	TGCTCCTTTGTTGGTATCG
pOASL	TCCCTGGGAAGAATGTGCAG	CCCTGGCAAGAGCATAGTGT
pMX-1	TAGGCAATCAGCCATACG	GTTGATGGTCTCCTGCTTAC
pPKR	GAGAAGGTAGAGCGTGAAG	CCAGCAACCGTAGTAGAG
pISG54	CTCAGAGGGTCAATGGAATTCC	CTGGCAAAGAGCCCTAAGGA
pIFITM3	GTCGTCTGGTCCCTGTTCAAC	GAGTAGGCGAAAGCCACGAA
pOAS	CTGTCGTTGGACGATGTATGCT	CAGCCGGGTCCAGAATCA
pIFN-α	GCCTCCTGCACCAGTTCTACA	TGCATGACACAGGCTTCCA
CP302L	TCTTTTGTGCAAGCATATACAGCTT	TGCACATCCTCCTTTGAAACAT
B646L	CCCAGGGGATAAAATGACTG	CACTGGTTCCCTCCACCGATA
DP96R	GAGAAACCAGCTGGAGCGAAT	ACAACATGGCACGTCATTTCC
pβ-Actin	CTCGATCATGAAGTGCGACG	GTGATCTCCTTCTGCATCCTGT

Table S1. List of primers used for real-time PCR.
